# Polymorphisms in the Vitamin D Receptor (VDR) and the Risk of Ovarian Cancer: A Meta-Analysis

**DOI:** 10.1371/journal.pone.0066716

**Published:** 2013-06-24

**Authors:** Yanling Liu, Chenglin Li, Peizhan Chen, Xiaoguang Li, Mian Li, He Guo, Jingquan Li, Ruiai Chu, Hui Wang

**Affiliations:** 1 Key Laboratory of Food Safety Research, Institute for Nutritional Sciences, Shanghai Institutes for Biological Sciences, Chinese Academy of Sciences, University of the Chinese Academy of Sciences, Shanghai, P. R. China; 2 Key Laboratory of Food Safety Risk Assessment, Ministry of Health, Beijing, P. R. China; MOE Key Laboratory of Environment and Health, School of Public Health, Tongji Medical College, Huazhong University of Science and Technology, China

## Abstract

The vitamin D receptor (VDR) principally mediates the anticancer activities of vitamin D. Various epidemiological studies have investigated the associations of VDR gene polymorphisms with ovarian cancer; however, the results have been inconclusive. In the current study, we evaluated, in a meta-analysis, the association of five common single nucleotide polymorphisms (SNPs) in the VDR gene (ApaI, BsmI, Cdx-2, FokI, and TaqI) with the risk of ovarian cancer. Six eligible studies, with a total of 4,107 cases and 6,661 controls, which evaluated the association of these variants and ovarian cancer risk, were identified from the MEDLINE and PubMed databases. The meta-analysis indicated that FokI was associated with an increased ovarian cancer risk, with a pooled odds ratio (OR) of 1.10 [95% confidence intervals (95% CI) = 1.00–1.20] for CT heterozygotes and 1.16 (95% CI = 1.02–1.30) for TT homozygotes relative to common CC carriers. Carriers of the T allele (also known as the f allele) showed an 11% (pooled OR = 1.11, 95% CI = 1.02–1.21; TT/CT vs. CC) increased risk of ovarian cancer relative to CC carriers. For FokI, no significant heterogeneity between the studies was found (I^2^ = 0%, P = 0.62 for the Q test). There was no statistically significant association between the other four variants (ApaI, BsmI, Cdx-2 and TaqI) and risk of ovarian cancer. These data indicate that the polymorphism FokI on the VDR is a susceptibility factor for ovarian cancer. Nevertheless, more studies are warranted to elucidate the underlying mechanisms of the VDR in development of ovarian cancer.

## Introduction

Ovarian cancer is one of the most lethal gynecological malignancies, with an estimated 225,500 new cases and 140,200 deaths worldwide annually [1]. Because of the low rate of early detection, the late clinical manifestation, and the lack of effective treatments, the 5-year survival rate for ovarian cancer patients is about 40% [1]. As 85% of the ovarian cancer cases are sporadic and 15% are familial, it has been hypothesized that both genetic and environmental factors may be involved in the development of ovarian cancer [2]. Factors including age, gravidity, tubal ligation, number of ovulatory cycles, and family history of ovarian cancer influence the ovarian cancer risk, as can lifestyle factors, including diet, physical activity, and exposure to carcinogenic chemicals [3].

Vitamin D is widely known for its classic roles in mineral metabolism and bone growth. Epidemiological and ecological studies have revealed the protective functions of vitamin D against ovarian, breast, colorectal, gastric, liver, prostate, and non-melanoma skin cancers [4], [5], [6]. In humans, the effects of 1α,25-dihydroxyvitamin D [1α,25-(OH)_2_D], the active form of vitamin D, are mainly mediated by the vitamin D receptor (VDR). As a transcriptional regulatory factor, the VDR is expressed in cells of the normal ovarian epithelium and ovarian tumor cells [7]. For the ovarian cancer cell line, OVCAR-3, 1α,25-(OH)_2_D reduces the proliferation induced by dihydrotestosterone through the VDR [8]. In ovarian cancer cells, 1α,25-(OH)_2_D3 leads to G2/M cell cycle arrest through a p53-independent induction of GADD45, which modulates tumor formation [9]. Vitamin D also has pleiotropic functions in the immune, neural, and endocrine systems [10], all of which are involved in the regulation of tumor growth and metastasis [11]. These data suggest that vitamin D could have preventive effects against ovarian cancer.

Since the VDR is a mediator of the vitamin D pathway, the association of VDR SNPs with ovarian cancer risk has been evaluated in various population studies [12], [13], [14], [15], [16], [17], [18], [19]. As reported for a case-control study, Caucasian T allele carriers for the variant FokI have a more than two-fold increased risk of ovarian cancer relative to homozygous CC carriers [15]. These results are consistent with the pooled results of five population-based, case-control studies, including 1,764 cases and 3,339 controls, which were performed by the Ovarian Cancer Association Consortium [14]. Nevertheless, several studies did not find such an association for FokI and risk for ovarian cancer [12], [16], [19]. The variant ApaI is reportedly associated with an increased risk of ovarian cancer among Caucasians but not for Japanese [15], and with an increased risk of ovarian cancer for African Americans but not for Caucasians [12]. Also in epidemiological studies, inconclusive results were found for other common variants, including BsmI, Cdx-2, and TaqI [12], [15], [16], [19], [20].

As the epidemiological studies that evaluated association of the VDR polymorphisms with risk of ovarian cancer found inconsistent results, we performed a systematic study to evaluate, with a meta-analysis, the associations of the common variants on the VDR and risk of ovarian cancer. Only FokI, which leads to an alteration of the VDR protein structure, was significantly associated with an increased risk; other common variants (BsmI, ApaI, TaqI, and Cdx-2) were not.

## Materials and Methods

### Literature Search

**Figure 1:**
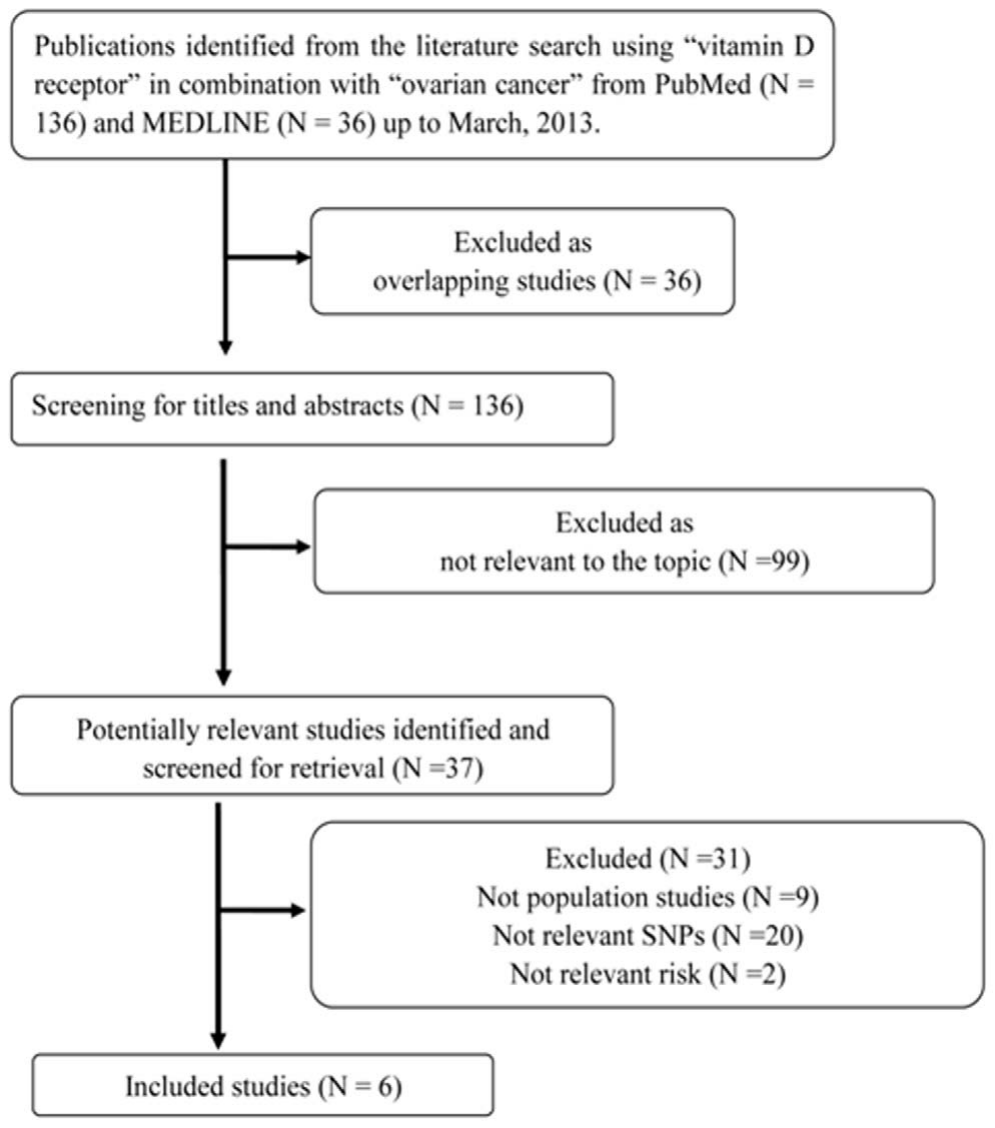
Flow chart for the literature search in the meta-analysis.

Two investigators (YL and CL) independently searched the MEDLINE and PubMed databases for eligible articles published prior to March, 2013. The terms “ovarian cancer” and “vitamin D receptor” were used to identify studies that assessed the association between VDR polymorphisms and the risk of ovarian cancer. The reference lists of the identified publications, including reviews, were checked to identify any missing study in the electronic database search. The working flow of the literature identification is shown in Figure 1.

### Inclusion and Exclusion Criteria

Studies to be included in the meta-analysis were required to meet the following criteria: 1) be a population- or hospital-based, case-control study or a prospective nested case-control study; 2) provided the frequencies of the variants, FokI, BsmI, ApaI, TaqI and Cdx-2 in the VDR in the cases and controls or provided sufficient data to calculate the risk estimates for the variants; and 3) reported in the English language. If overlapping populations were identified between studies, only the most complete one was included in the meta-analysis.

### Data Extraction

From each report, the following data were extracted: the last name of the first author, the publication year, the region in which the study was performed, the ethnicity of the participants, the study design (source of controls), the method used for genotyping of the polymorphisms, the sample size, and the genotype distribution for the participants.

### Statistical Analysis

The pooled ORs with their 95% CIs were calculated to assess the association between polymorphisms and the risk of ovarian cancer under the additive, dominant, and recessive genetic models. The Chi-square test was used to determine if the identified study was in accordance with Hardy-Weinberg equilibrium (HWE) for the genotype distribution in the control group. The standard inverse variance weighting method was used to calculate the pooled ORs and 95% CIs under the fixed-effects model, and the DerSimonian-Laird method was used to calculate the pooled estimates under the random-effects model. The heterogeneity between the studies was evaluated with the Q-test and the I^2^ statistic. Heterogeneity between the studies was considered to be significant when P<0.05 for Q-tests or when I^2^ was more than 25%. Sensitivity analyses were performed to assess the stability of the final results, and the influence of individual studies on the pooled estimate risk was tested by deleting each single study from the meta-analysis. Publication bias was measured by funnel plots and further evaluated with the Egger’s linear regression test [21], [22], [23]. All statistical analyses were performed with R software and the Meta package for R (www.r-project.org).Two-sided P<0.05 was considered statistically significant.

## Results

### Characteristics of Selected Studies

136 published reports before March, 2013 were identified in the database search. Of them, six studies met the inclusion criteria for our meta-analysis (Figure 1). Subgroup studies from three reports that contained more than one study subgroup were recognized as individual studies [14], [18], [19]. Since the report by Lurie et al. [14] had an overlapping population with another study [15], the subgroup with the smaller sample size was excluded from the analysis. A total of 4,107 cases and 6,661 controls from 11 subgroup studies that reported the association between the 5 common variants (ApaI, BsmI, Cdx-2, FokI and TaqI) and ovarian cancer risk were included in the meta-analysis. The characteristics of the identified studies are shown in Table 1.

**Table 1:** Characteristics of publications identified for the meta-analysis.

Study (Author, year)		Study Design	Study Region		Ethnicity/Subgroup	Sample Size (Case/Control)	Genotype Distribution (Case/Control)								Genotyping Method	Ref	
							FokI (rs2228570/rs10735810)										
							CC		CT			TT					
Lurie, 2007		PB	Hawaii, USA		Caucasian	71/144	16/58		44/64			11/22			Taqman	15	
		PB	Hawaii, USA		Japanese	93/172	37/74		48/80			8/18			Taqman	15	
Clendenen, 2008		NCC	New York, USA		Caucasian	71/125	27/51		33/57			11/17			PCR-RFLP	19	
		NCC	Northern Sweden		Caucasian	97/196	39/69		42/95			16/32			PCR-RFLP	19	
Tworoger, 2009		PB	Massachusetts/New Hampshire, USA		Mixed (NECC)	1104/1136	409/450		502/511			193/175			Taqman	18	
		NCC	USA		Mixed (NHS/NHSII/WHS)	288/757	98/304		141/340			49/113			Taqman	18	
Lurie, 2011		PB	Denmark		Caucasian (MALOVA)	424/1183	159/475		208/545			57/163			Taqman	14	
		PB	UK		Caucasian (SEARCH)	813/1224	296/484		406/552			111/188			Taqman	14	
		PB	California, USA		Caucasian (GEOCS)	269/365	104/146		116/176			49/43			Taqman	14	
		PB	UK		Caucasian (UKOPS)	258/567	101/220		115/281			42/66			Taqman	14	
Grant, 2013		PB	North Carolina, USA		Caucasian	396/531	53/72		185/256			158/203			Taqman	12	
		PB	North Carolina, USA		African American	55/79	4/3		16/22			35/54			Taqman	12	
Mostowska, 2013		PB	Wielkopolska, Poland		Caucasian	168/182	47/51		83/102			38/29			PCR-RFLP	16	
							**ApaI (rs7975232)**										
							**TT**	**TG**			**GG**						
Lurie, 2007		PB	Hawaii, USA	Caucasian		70/139	22/34	37/72			11/33			Taqman		15	
		PB	Hawaii, USA	Japanese		93/168	8/14	47/79			38/75			Taqman		15	
Clendenen, 2008		NCC	New York, USA	Caucasian		71/124	25/37	37/58			9/29			PCR-RFLP		19	
		NCC	Northern Sweden	Caucasian		97/196	30/49	43/94			24/53			PCR-RFLP		19	
Grant, 2013		PB	North Carolina, USA	Caucasian		392/532	105/148	204/261			83/123			Taqman		12	
		PB	North Carolina, USA	African American		94/154	35/68	44/72			15/14			Taqman		12	
							BsmI (rs1544410)										
							GG			GA			AA				
Lurie, 2007		PB	Hawaii, USA	Caucasian		71/144	26/48			30/70			15/26	Taqman		15	
		PB	Hawaii, USA	Japanese		94/171	72/137			19/28			3/6	Taqman		15	
Clendenen, 2008		NCC	New York, USA	Caucasian		70/123	28/50			36/61			6/12	PCR-RFLP		19	
		NCC	Northern Sweden	Caucasian		98/195	39/86			40/75			19/34	PCR-RFLP		19	
Tworoger, 2009		PB	Massachusetts/New Hampshire, USA	Mixed (NECC)		1113/1151	409/430			521/518			183/203	Taqman		18	
		NCC	USA	Mixed (NHS/NHSII/WHS)		278/734	94/267			143/353			41/114	Taqman		18	
Grant, 2013		PB	North Carolina, USA	Caucasian		385/523	129/192			197/244			59/87	Taqman		12	
		PB	North Carolina, USA	African American		54/78	34/38			17/38			3/2	Taqman		12	
Mostowska, 2013		PB	Wielkopolska, Poland	Caucasian		168/182	60/87			91/78			17/17	PCR-RFLP		16	
							**Cdx-2 (rs11568820)**										
							**GG**			**GA**			**AA**				
Lurie, 2007	PB		Hawaii, USA	Caucasian		70/145	44/95			21/44			5/6	Taqman		15	
	PB		Hawaii, USA	Japanese		92/171	36/45			41/99			15/27	Taqman		15	
Tworoger, 2009	PB		Massachusetts/New Hampshire, USA	Mixed (NECC)		1120/1158	670/746			399/356			51/56	Taqman		18	
	NCC		USA	Mixed (NHS/NHSII/WHS)		285/752	179/496			92/220			14/36	Taqman		18	
							**TaqI (rs731236)**										
							**TT**			**TC**			**CC**				
Lurie, 2007	PB		Hawaii, USA	Caucasian		72/146	26/48			30/77			16/21	Taqman		15	
	PB		Hawaii, USA	Japanese		93/173	73/138			17/31			3/4	Taqman		15	
Clendenen, 2008	NCC		New York, USA	Caucasian		71/124	27/49			35/58			9/17	PCR-RFLP		19	
	NCC		Northern Sweden	Caucasian		98/196	41/88			40/75			17/33	PCR-RFLP		19	
Grant, 2013	PB		North Carolina, USA	Caucasian		395/530	131/198			205/243			59/89	Taqman		12	
	PB		North Carolina, USA	African American		55/79	32/43			18/31			5/5	Taqman		12	

**Abbreviations:** PB, population-based case-control study; NCC, nested case-control study; PCR-RFLP, polymerase chain reaction-restriction fragment length polymorphism; NECC, new England case-control study; NHS, nurses’ health study; WHS, women’s health study; MALOVA, malignant ovarian cancer study; SEARCH, studies of epidemiology and risk factors in cancer heredity: ovarian cancer study; GEOCS, genetic epidemiology of ovarian cancer study; HAW, Hawaii ovarian cancer study; UKOPS, united kingdom ovarian cancer population study.

### FokI (rs2228570 or rs10735810) and Ovarian Cancer Risk

**Figure 2:**
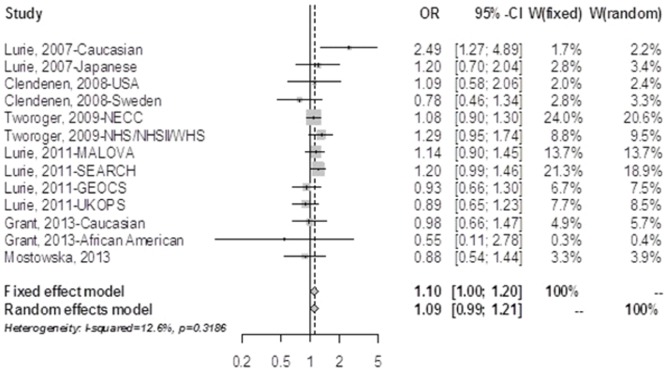
Forest plot for the association between variant FokI in the VDR and risk of ovarian cancer (CT vs. CC). NECC, new England case-control study; NHS, nurses’ health study; WHS, women’s health study; MALOVA, malignant ovarian cancer study; SEARCH, studies of epidemiology and risk factors in cancer heredity: ovarian cancer study; GEOCS, genetic epidemiology of ovarian cancer study; UKOPS, united kingdom ovarian cancer population study.

**Figure 3:**
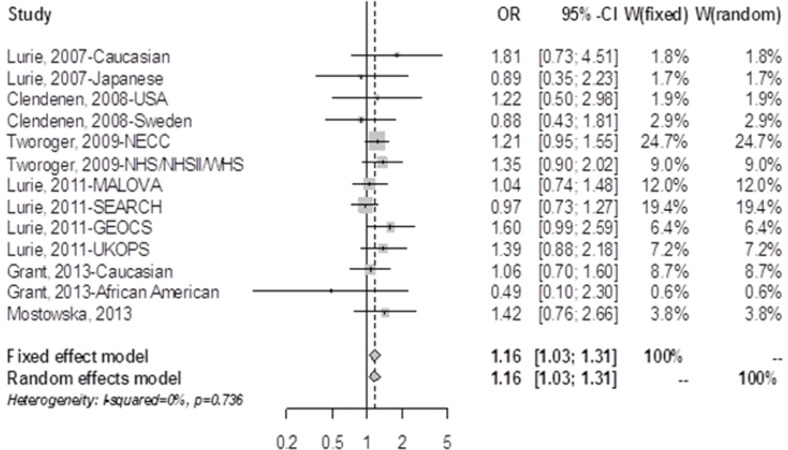
Forest plot for the association between variant FokI in the VDR and risk of ovarian cancer (TT vs. CC). NECC, new England case-control study; NHS, nurses’ health study; WHS, women’s health study; MALOVA, malignant ovarian cancer study; SEARCH, studies of epidemiology and risk factors in cancer heredity: ovarian cancer study; GEOCS, genetic epidemiology of ovarian cancer study; UKOPS, united kingdom ovarian cancer population study.

As established from the literature search, all 11 subgroup studies, with 4,107 cases and 6,661 controls, evaluated the association between FokI and risk of ovarian cancer. None of the subgroup studies departed from the Hardy-Weinberg equilibrium for the allele T distribution in the controls. The meta-analysis indicated that FokI was significantly associated with an increased risk of ovarian cancer. As determined under the fixed-effect model, the pooled OR for CT carriers was 1.10 (95% CI = 1.00–1.20) and 1.16 (95% CI = 1.03–1.31) for the TT carriers relative to CC carriers (Figure 2 and Figure 3). The dominant genetic model also suggested that carriers of the T allele were associated with an 11% increased risk for ovarian cancer (pooled OR = 1.11, 95% CI = 1.02–1.21). The recessive genetic model indicated that carriers of homozygote TT showed a marginal 10% increased risk of ovarian cancer compared to those of TC or CC carriers (pooled OR = 1.10, 95% CI = 0.99–1.22). Similar results were found under the random-effects model as no significant heterogeneity between the studies was found for any meta-analysis of the variant (Table 2). Sensitivity analysis suggested that no single subgroup study significantly affected the overall estimates of the association between FokI and ovarian cancer. The funnel plot and the Egger’s test showed no significant publication bias for the identified subgroups. When we restricted the studies that were conducted in the Caucasians (3,959 cases and 6,410 controls), we found a significant association for the allele T carriers (pooled OR = 1.12, 95% CI = 1.02–1.22) when compared to the homozygotes CC carriers with no significant heterogeneity between the studies were identified.

**Table 2:** Associations between VDR polymorphisms (ApaI, BsmI, Cdx-2, FokI, TaqI) and the risk of ovarian cancer under different genetic models.

SNP	Genetic model	Fixed effects model	Random effects model	Heterogeneity				Publication bias
		OR (95% CI)	OR (95% CI)	Q/d.f.	I^2^		P	P
FokI	CT vs. CC	1.10 [1.00–1.20]	1.09 [0.99–1.21]	13.7/12		13%	0.32	0.61
	TT vs. CC	1.16 [1.03–1.31]	1.16 [1.03–1.31]	8.61/12		0%	0.74	0.93
	Dominant	1.11 [1.02–1.21]	1.11 [1.02–1.21]	9.90/12		0%	0.62	0.70
	Recessive	1.10 [0.99–1.22]	1.10 [0.99–1.22]	12.3/12		2%	0.42	0.71
ApaI	TG vs. TT	1.00 [0.81–1.24]	1.00 [0.81–1.24]	2.21/5		0%	0.82	0.33
	GG vs. TT	0.87 [0.67–1.14]	0.85 [0.59–1.22]	7.97/5		37%	0.16	0.67
	Dominant	0.96 [0.79–1.18]	0.96 [0.79–1.18]	4.05/5		0%	0.39	0.39
	Recessive	0.87 [0.70–1.08]	0.86 [0.66–1.13]	6.96/5		28%	0.22	0.82
BsmI	GA vs. GG	1.11 [0.99–1.25]	1.12 [0.96–1.29]	9.89/8		19%	0.27	0.71
	AA vs. GG	1.01 [0.86–1.20]	1.01 [0.86–1.20]	1.86/8		0%	0.99	0.09
	Dominant	1.09 [0.97–1.22]	1.09 [0.97–1.23]	8.54/8		6%	0.38	0.93
	Recessive	0.96 [0.82–1.11]	0.96 [0.82–1.11]	1.97/8		0%	0.98	0.06
Cdx-2	GA vs. GG	1.15 [1.00–1.33]	1.02 [0.75–1.38]	8.48/3		65%	0.05	0.22
	AA vs. GG	1.00 [0.74–1.35]	1.00 [0.74–1.35]	1.78/3		0%	0.62	0.75
	Dominant	1.14 [0.99–1.30]	1.04 [0.79–1.36]	7.38/3		59%	0.06	0.29
	Recessive	1.01 [0.76–1.35]	1.01 [0.76–1.35]	0.97/3		0%	0.81	0.06
TaqI	TC vs. TT	1.10 [0.90–1.35]	1.10 [0.90–1.35]	3.60/5		0%	0.61	0.05
	CC vs. TT	1.09 [0.82–1.45]	1.09 [0.82–1.45]	0.84/5		0%	0.97	0.14
	Dominant	1.10 [0.91–1.33]	1.10 [0.91–1.33]	1.54/5		0%	0.91	0.04
	Recessive	1.02 [0.78–1.33]	1.02 [0.78–1.33]	3.24/5		0%	0.66	0.16

### ApaI (rs7975232) and Ovarian Cancer Risk

Six subgroup studies from 3 reports, which included a total of 817 cases and 1,313 controls, evaluated the association for the variant ApaI (rs7975232) and risk for ovarian cancer (Table 1). None of the subgroup studies departed from the Hardy-Weinberg equilibrium for the allele G distribution in the controls. As determined with the fixed-effect model, there was no significant association for the variant and risk of ovarian cancer; relative to TT carriers, the pooled OR was 1.00 (95% CI = 0.81–1.24) for TG carriers and 0.87 (95% CI = 0.67–1.14) for GG carriers. Under the dominant genetic model, there was also no significant association for carriers of the G allele and the risk of ovarian cancer (pooled OR = 0.96, 95% CI = 0.79–1.18), as determined with the fixed-effect model. When we restricted to the Caucasians, no significant association for the variant and ovarian cancer risk was found (pooled OR = 0.91, 95% CI = 0.73–1.14; 630 cases and 991 controls). No significant heterogeneity between the studies was detected, and the Egger’s test indicated no significant publication bias for the subgroup studies (Table 2).

### BsmI (rs1544410) and Ovarian Cancer Risk

Nine subgroup studies from 5 publications, including a total of 2,332 cases and 3,301 controls, concerned the association of BsmI (rs1544410) and risk of ovarian cancer. Of these, the genotype distribution in the controls from four subgroup studies, including the Japanese subgroup by Lurie et al. [15], Northern Sweden subgroup by Clendenen et al. [19], the New England Case-Control (NECC) subgroup by Tworoger et al. [18], and the African American subgroup by Grant et al. [12], was departed from the Hardy-Weinberg equilibrium (P<0.05) as indicated by Chi-square tests. The fixed-effect model suggested no significant association between the variant and ovarian cancer risk (GA vs. GG: pooled OR = 1.11, 95% CI = 0.99–1.25; AA vs. GG: pooled OR = 1.01, 95% CI = 0.86–1.20), as under the dominant genetic model (pooled OR = 1.09, 95% CI = 0.97–1.22; AA/GA vs. GG). After excluding the subgroup conducted in the African American population performed by Grant et al. [12], which contributed most to the heterogeneity between the studies, the pooled estimate suggested that carriers of the A allele showed a significantly increased risk of ovarian cancer (pooled OR = 1.14, 95% CI = 1.01–1.28) relative to GG carriers. No significant publication bias was found for the meta-analysis (Table 2). When we restricted the studies to Caucasians, we also found no significant association for the variant with ovarian cancer risk (pooled OR = 1.10, 95% CI = 0.98–1.24; 2,183 cases and 3,052 controls) with no significant heterogeneity between the studies were identified (Q = 4.94, df = 6, P = 0.551; I^2^ = 0%).

### Cdx-2 (rs11568820) and Ovarian Cancer Risk

Two reports with 4 subgroup studies and with a total of 1,567 cases and 2,226 controls evaluated the association of Cdx-2 and ovarian cancer risk [15], [18]. Of these, subgroup studies conducted with a Japanese subgroup by Lurie et al. [15] and with the NECC subgroup by Tworoger et al. [18], were departed from the Hardy-Weinberg equilibrium (P<0.05). With the random-effects model, the meta-analysis suggested no significant association for the variant and the risk of ovarian cancer; the pooled OR was 1.02 (95% CI = 0.75–1.38) for GA and 1.00 (95% CI = 0.74–1.35) for AA carriers relative to the common GG carriers. The dominant genetic model also indicated that the variant was not associated with risk of ovarian cancer, and no significant heterogeneity between the studies was found (Table 2). The Egger’s test found no publication bias for the meta-analysis (Table 2). When we restricted the studies to Caucasians, we found that the variant was associated with the ovarian cancer risk (pooled OR = 1.19, 95% CI = 1.04–1.37; 1,475 cases and 2,055 controls) with no significant heterogeneity between the studies were identified (Q = 0.16, df = 2, P = 0.922; I^2^ = 0%).

### TaqI (rs731236) and Ovarian Cancer Risk

From the literature search, 6 subgroup studies from 3 reports that considered 784 cases and 1,248 controls evaluated the association between TaqI and risk of ovarian cancer. The Northern Sweden subgroup, performed by Clendenen et al. [19], departed from the Hardy-Weinberg equilibrium (P<0.05). Our meta-analysis indicated that the variant had no significant effect on the risk of ovarian cancer. The pooled OR was 1.10 (95% CI = 0.90–1.35) for TC carriers and 1.09 (95% CI = 0.82–1.45) for CC carriers relative to TT carriers. The fixed-effect model and the random-effects model found a null association for the variant and risk of ovarian cancer under the dominant genetic model (pooled OR = 1.10, 95% CI = 0.91–1.33). There was no significant heterogeneity between studies included in the meta-analysis. However, there was a marginal publication bias under the dominant genetic model (P = 0.04, Table 2). Sensitivity tests showed that no single study greatly influenced the estimates of overall risk. When restricted the studies performed in the Caucasians, we also noticed no significant association between the variant and the ovarian cancer (pooled OR = 1.13, 95% CI = 0.91–1.39; 636 cases and 996 controls).

## Discussion

The present meta-analysis evaluated the association of five common VDR polymorphisms (ApaI, BsmI, Cdx-2, FokI and TaqI) and the risk of ovarian cancer. The variant FokI on the VDR was associated with the risk of ovarian cancer, but there was no significant association for the other four variants. The data suggest that a dysfunction of vitamin D and its downstream signaling pathways are involved in the development of ovarian cancer.

FokI, located at the translation start site of the VDR gene, results in alteration of the VDR protein sequence, which is three amino acids longer for carriers of the T (also known as the f allele for FokI) allele than for carriers of the C allele (also known as the F allele for FokI) [24], [25]. As determined by epidemiological studies, the variant may affect the susceptibility to a variety of cancers. In a previous meta-analysis, populations with FokI f allele were associated with a 14% increased risk (pooled OR = 1.14, 95% CI = 1.03–1.27) of breast cancer and a 30% increased risk (pooled OR = 1.30, 95% CI = 1.04–1.61) of skin cancer; however, there was no significant association of the variant and prostate or colorectal cancer [26]. Another meta-analysis performed by Yin et al. updated the epidemiological studies with a total of 6,736 cases and 7,325 controls found that FokI was not significantly associated with risk for prostate cancer; however, under the dominant genetic model, there was an increased risk of prostate cancer in Caucasians (pooled OR = 1.08, 95% CI = 1.00–1.17) [27]. Thus, the altered biological activities of this VDR variant may lead to susceptibility to various types of cancer. For the current study, we have included a total of 4,107 cases and 6,661 controls from 6 eligibility studies and found that allele T confer an increased risk for ovarian cancer. The data suggested that dysfunction of vitamin D signaling pathways may be involved in the ovarian cancer carcinogenesis. As the FokI T allele results in three amino acids longer VDR protein than the C allele [24], it has been reported that the longer VDR protein is less responsive to 1α,25-(OH)_2_D and has lower transcription activity [28], [29], [30]. Carriers of the T allele may have a lower response ability for the protective effects of vitamin D. Previous studies suggested that the variant could modify the transcriptional factor activities of VDR on various target genes including genes involved in the immune system such as NF-kB and AP-1 [31]. Etten et al. reported that longer VDR (allele T or F carriers) resulted in a lower NF-kB transcriptional activation and further leaded to a reduced IL-12 expression and weaker immunity responses [31]. It was widely accepted that the immune system play an important roles in ovarian cancer development and progression. The C to T transition leads to longer VDR protein may be resulted in the reduced immunity response, which may be contributed to the ovarian cancer susceptibility.

Located at the 3′-UTR of the VDR gene, BsmI (rs1544410), ApaI (rs7975232), and TaqI (rs731236) are SNPs that are linked with each other with high linkage disequilibrium. These SNPs do not affect the VDR protein structure but may affect the stability of VDR mRNA or may be in high linkage disequilibrium with other functional SNPs [25]. As determined in a larger population based case-control study, the BsmI B allele was significantly associated with an increased risk of melanoma [32]; however, the results were different from previous meta-analyses, which suggested that the B allele was a protective factor for melanoma [32]. BsmI was significantly associated with a decreased risk of colorectal cancer in a meta-analysis that included 10,083 cases and 11,242 controls (pooled OR = 0.87, 95% CI = 0.80–0.94 for BB vs. bb; pooled OR = 0.90, 95% CI = 0.84–0.97 for BB vs. Bb/bb), indicating a protective effect of the B allele [33]. There was also a reduced risk of prostate cancer for carriers of the B allele of BsmI (pooled OR = 0.83, 95% CI = 0.69–0.99) [26]. TaqI was reported to be associated with risk of prostate cancer by Yin et al., based on case-control study including 4,054 cases and 5,069 controls [27]. A recent meta-analysis conducted by Bai et al., however, did not find a significant association for TaqI and risk of colorectal cancer [33]. Nevertheless, variant ApaI was significantly associated with risk of colorectal cancer [33]. Our meta-analysis found no significant association for these three variants and risk of ovarian cancer. The inconsistent results from the epidemiological studies regarding the associations of the variants with risk of different cancers could be due to different genetic backgrounds of the cancers or to different modes of action of vitamin D between tissues. 1α,25-(OH)_2_D influences gene transcription by binding to specific DNA sequences of the gene promoter, which are promoter-specific and cell-specific [10]. The variability in DNA-responsive sequences, VDR isoforms, cell-specific phosphorylations, and co-regulators in different tissues could alter the binding capacity of the VDR for its target sequences. Moreover, different VDR-DNA complexes could lead to conformation changes of the VDR and result in specific interactions with cell-specific transcription factors. These could be the underlying mechanisms for the cell-specific functions of 1α,25-(OH)_2_D [25], [34] and could lead to diverse associations between the VDR polymorphisms and the risk of cancer of different tissues.

In the case of Cdx-2, which is located in the promoter region of the VDR gene, a G to A sequence variation affects the binding of this intestinal-specific transcription factor CDX. The A allele is more active by binding to the transcription factor, resulting in greater transcription activity [35]. Bai et al. did not find a significant association for the variant and colorectal cancer risk based on a meta-analysis based on 2,639 cases and 2,939 controls [33]. Yin et al. also found a null association between Cdx-2 and risk of prostate cancer with a case-control study including 2,058 cases and 2,128 controls [27]. These results are consistent with a report by John et al. [36]. We found no significant association for the variant and ovarian cancer risk for the pooled estimate of overall studies; however, when we restricted the studies to Caucasians, a significant increased risk was found for the allele A carriers (pooled OR = 1.19, 95% CI = 1.04–1.37) indicated that the variant could be a risk factor for ovarian cancer. It should be noticed that data regarding the association for Cdx-2 and ovarian cancer risk are limited; more studies are warranted to reveal the biological implications of this variant on cancer risk.

For the current meta-analysis, several limitations should be taken into consideration. First, most of the patients were Caucasians, which may limit the general application of our results. Second, the sample size was relatively small, and all data were from case-control studies. The null association for the variants, BsmI, ApaI, TaqI, and Cdx-2, could be due to the small sample size, which provides low statistical power to detect the association for the variants. More studies with larger sample sizes are needed to obtain more reliable results. Third, we were not able to take into consideration other factors, such as circulating vitamin D levels, outdoor activity, sun exposure, disease stage, and vitamin D and calcium intake, that may modify the association of VDR variants and risk of ovarian cancer [37], [38], [39], [40]. It is possible that the interactions between VDR polymorphisms and these factors are involved in the development of ovarian cancer, and the association for the variants and risk of ovarian cancer may be modified by these factors. Finally, several subgroup studies departed from the Hardy-Weinberg equilibrium (P<0.05), which may have led to a bias for the overall estimates of the meta-analysis.

In conclusion, our meta-analysis indicates that FokI is associated with an increased risk of ovarian cancer and that the VDR can be a preventive target for ovarian cancer. Nevertheless, more studies are warranted to confirm the results and to establish the underlying molecular mechanisms that are involved.
